# Associations between sports participation, adiposity and obesity-related health behaviors in Australian adolescents

**DOI:** 10.1186/1479-5868-10-113

**Published:** 2013-10-02

**Authors:** Stewart A Vella, Dylan P Cliff, Anthony D Okely, Maree L Scully, Belinda C Morley

**Affiliations:** 1 Interdisciplinary Educational Research Institute, Faculty of Social Sciences, Northfields Avenue, University of Wollongong, Wollongong 2522, Australia; 2 Centre for Behavioural Research in Cancer, Cancer Council Victoria, 1 Rathdowne Street, Carlton 3053, Australia

**Keywords:** Overweight, Dietary behaviors, Physical activity, Screen time, Public health

## Abstract

**Background:**

The purpose of this study was to examine the relationship between organized sports participation, weight status, physical activity, screen time, and important food habits in a large nationally representative sample of Australian adolescents.

**Methods:**

Nationally representative cross-sectional study of 12,188 adolescents from 238 secondary schools aged between 12 and 17 years (14.47 ± 1.25 y, 53% male, 23% overweight/obese). Participation in organized sports, compliance with national physical activity, screen time, and fruit and vegetable consumption guidelines, and consumption of sugar-sweetened beverages and high-fat foods were self-reported. Weight status and adiposity (BMI, waist circumference) were measured.

**Results:**

Organized sports participation was higher among males and those residing in rural/remote areas. Underweight adolescents reported the lowest levels of participation. Higher levels of participation were associated with an increased likelihood of complying with national physical activity (OR = 2.07 [1.67-2.58]), screen time (OR = 1.48 [1.19-1.84]), and fruit and vegetable consumption guidelines (OR = 1.32 [1.05-1.67]). There was no association between organized sport participation and weight status, adiposity, consumption of sugar-sweetened beverages or high-fat foods.

**Conclusions:**

Participation in organized sports was associated with a greater likelihood to engage in a cluster of health behaviors, including meeting physical activity guidelines, electronic screen time recommendations, and fruit and vegetable consumption guidelines. However, participation in organized sports was not associated with unhealthy dietary behaviors including the consumption of sugar-sweetened beverages and high-fat foods. There is no association between participation in organized sports and likelihood to be overweight or obese. The role of sports in promoting healthy weight and energy balance is unclear.

## Background

Approximately 25% of adolescents in developed countries are overweight or obese [1,2]. Obesity is now recognized as one of the most pressing public health problems [3], reducing life expectancy and quality of life [4]. The World Health Organization, United Nations, and the International Olympic Committee have advocated that organized sports have an important role to play in reducing the worldwide burden of obesity in childhood and adolescence [5-7]. For example, one study reported that participation in at least two organized sports per year may reduce the prevalence of adolescent obesity by up to 26% [8]. This may be due to its association with key obesity-related behaviors such as increased levels of physical activity and healthy dietary habits [9]. However, a recent systematic review found that there was no clear association between youth sports participation and weight status [10]. Youth sports participants were more physically active and consumed greater amounts of fruit and vegetables, but also consumed greater amounts of fast food, sugar-sweetened beverages, and overall calories when compared to nonparticipants [10]. Furthermore, few of the studies reviewed used nationally-representative samples, and none were conducted in Australia.

Organized sports have a prominent place in the Australian identity [11] and typically two-thirds of all adolescents participate in at least one organized sport in any given year [12,13]. The purpose of this study was to examine the relationship between organized sports participation, weight status, physical activity, screen time, and important food habits in a large nationally representative sample of Australian adolescents. Importantly, this study also addressed the limitation that “Few datasets describe the population prevalence of participation in sports by total min per week, which makes the accurate estimation of the population-level health benefit attributable to sport impossible” [14]; pg. 59. As a corollary, this study investigated the associations between adolescent organized sports participation in min per week, and compliance with national physical activity and electronic screen time guidelines, selected weight-related eating behaviors, weight status and adiposity in a nationally-representative sample of Australian adolescents.

## Methods

### Study design and participants

Data were obtained from the Australian National Secondary Students’ Diet and Activity (NaSSDA) survey, conducted from May 2009 to April 2010. The NaSSDA survey is a nationally representative survey of Australian secondary students in Grades 8 through 11 (ages 12–17 yrs) established to monitor trends in obesity prevalence, diet, and activity triennially. The survey methods have been described in detail elsewhere [15]. Briefly, a stratified, two-stage probability sampling procedure was used, with schools (government, Catholic and independent) proportionally randomly selected at the first stage of sampling and classes selected from within schools at the second stage. Written informed parent and student consent was required for each component of the study. Approval for the study was given by Cancer Council Victoria’s Human Research Ethics Committee, all relevant education authorities, and school principals. The characteristics of the study sample, including sex, school grade, socio-economic position (SEP), geographic location, and weight status have been previously reported [15], along with the prevalence and socio-demographic distribution of eating, physical activity and sedentary behaviors [16].

### Measures

An online questionnaire assessing organized sports participation, compliance with physical activity, screen-based entertainment and fruit and vegetable consumption guidelines, as well as consumption of high-fat foods and sugar-sweetened beverages, was administered to students at school within their class group.

#### Organized sports participation

The *Adolescent Physical Activity Recall Questionnaire* (APARQ) [17] was used to assess average weekly participation in organized sports; a component of APARQ with good reliability and validity [17]. Organized sports and games were defined as those which involve competition, coaching or training sessions, and are organized by adults. Examples include playing on a sports team, gymnastics or dancing classes, swimming squads, or attending fitness classes. Students were asked to think about a usual week during the summer and winter school terms and to report, separately, their participation in all organized sports and games. For each sport, students reported the weekly frequency of participation, and the duration of participation each time, including training. Students’ weekly participation was calculated by averaging the number of total minutes reported per week over summer and winter school terms. Values greater than 1680 mins.wk^-1^ (equal to 4 hrs of organized sports participation per day) were considered implausible and replaced with the maximum plausible value of 1680 mins/wk. Students were categorized into quintiles for logistic regression analyses. Values of simple kappa for the organized sport component of the APARQ ranged from 0.44–0.89 and 0.65–0.87 for summer and winter school terms, respectively [17].

#### Physical activity guidelines

To assess compliance with Australian physical activity guidelines [18], students were asked “Over the past 7 days, on how many days were you active for 60 minutes or more per day?” Responses were categorized into those who reported 60 minutes of physical activity on all 7 days of the week (meeting guidelines) or not. This screening measure has established validity in this age group [19].

#### Screen-based entertainment guidelines

Students were asked to indicate how long they spent: (i) watching TV; (ii) watching videos/DVDs; (iii) playing video games; and (iv) using the computer for fun, on a usual school day and weekend day. Students’ weighted average daily time spent in small screen recreation was calculated by ([school day responses × 5] + [weekened day responses  × 2]/7) and categorized into ≤ 2 hours or > 2 hours per day to assess compliance with Australian screen-based entertainment guidelines [18]. This instrument has established reliability in this age group [20].

#### Dietary behaviors

To assess students’ eating habits, items from a short validated dietary questionnaire were used(18). Students indicated how many serves of (i) vegetables (excluding potatoes, hot chips and fried potato) and (ii) fruit (excluding fruit juice) they usually ate each day (including all fresh, dried, frozen and tinned varieties). A serve of vegetables was defined as ½ cup of cooked vegetables or 1 cup of salad vegetables, while a serve of fruit was specified as 1 medium piece, 2 small pieces or 1 cup of diced fruit [21]. Students were categorized as meeting the national recommendations if they reported eating four or more daily serves of vegetables and three or more daily serves of fruit [22].

To assess consumption of sugar-sweetened beverages, students were asked “How much soft drink (like Coke, lemonade), cordials or sports drinks (like Gatorade) do you usually drink each day or week?” Students were dichotomised into those who consumed sugar-sweetened beverages on a daily basis or not. High-fat food consumption was assessed by asking “How often do you have meals or snacks such as burgers, pizza, chicken or chips from places like McDonalds, Hungry Jacks/Burger King, Pizza Hut, KFC, Red Rooster or local take away food places?” Students were dichotomised into those who consumed high-fat food on a weekly basis or not. Both items have been shown to discriminate between different categories of intake among students within this sample [23].

#### Anthropometric measures

All anthropometric measures (height, weight and waist circumference) were taken by trained research assistants in a confidential setting following standardised protocols [24]. Height was measured using portable stadiometers (Model IP0955; Invicta Plastics, Leicester, UK), and weight was measured using digital scales (Model UM051; Tanita, Tokyo, Japan). Waist circumference was measured using an inelastic tape measure. Body Mass Index (BMI; kg/m^2^) and waist-to-height ratio were calculated. Students were classified into weight categories (underweight, healthy weight, overweight, or obese) using International Obesity Task Force definitions [25].

#### Covariates

Students’ sex, age and home postcode were recorded. Based on the students’ home postcode, a measure of SEP was determined according to the Socio-Economic Indexes for Areas (SEIFA) Index of Relative Socio-Economic Disadvantage [26]. Students were categorised into low SEP (quintiles 1 and 2), medium SEP (quintiles 3 and 4), and high SEP (quintile 5) groups. Home postcode was used to classify the geographic location of students into metropolitan or rural/regional areas according to the Rural, Remote and Metropolitan Area Classification [27]. Further, cultural background was measured using the item “What is the main language spoken at home?”. Responses were collapsed into one of five categories described by the Australian Bureau of Statistics: English; European; Asian; Middle-Eastern; or, Other.

### Statistical analyses

Using Stata SE version 11 (StataCorp, Texas), data were weighted by state, education sector, school grade and sex to ensure that the sample reflected the population distribution [28]. Unadjusted and adjusted logistic regression analyses examined the associations between students’ organized sport participation and their weight status, adiposity, eating habits, and likelihood of meeting physical activity and screen-based entertainment guidelines. Adjusted models controlled for sex, age, SEP, cultural background, geographic location, school level clustering and school type. Simple and multiple linear regressions were used to analyse the relationship between participation in organized sports and waist-to-height ratio and waist circumference. Due to the large sample size, a conservative level of statistical significance (*p* < 0.01 for logistic and linear regressions, *p* < 0.05 for interaction effects) was applied.

## Results

### Participants

Two-hundred and thirty-eight schools (response rate 39%) were recruited, with data collected from 14,066 students (response rate 54%). Students were excluded if they were not aged 12–17 yrs (53 participants), did not complete >33% of the survey questions (223 participants), did not complete the anthropometric measures (1515 participants), or no SEP index value was available for the postcode (87 participants). Thus, a total of 12,188 participants (87%) were included in these analyses. Females (15% excluded) were more likely than males (7%) to be excluded, primarily due to non-completion of anthropometric measures. There were no other differences between those who were excluded from the study and those that were not. Quintiles of organized sports participation were as follows: Quintile 1 (0–60 mins.wk^-1^), quintile 2 (61–210 mins.wk^-1^), quintile 3 (211–405 mins.wk^-1^), quintile 4 (406–750 mins.wk^-1^), and quintile 5 (751–1680 mins.wk^-1^). Table 1 displays the characteristics of the sample.

**Table 1:** Characteristics of Study Sample and Mean Level of Organized Sports Participation

**Characteristics (missing data)**	**N (%)**	**Mean OSP**^**1 **^**(SD)**	
Sex (0)			
Male	6,460 (53)	505 (481)^*a*^	*t* = 15.91, *p* < .001
Female	5,728 (47)	373 (423)^*b*^	
Age (0)			
12	370 (3)	425 (472)	*F =* 1.90*, p =* .091
13	2,726 (22)	442 (451)	
14	3,348 (28)	451 (460)	
15	2,928 (24)	457 (466)	
16	2,151 (18)	422 (460)	
17	665 (6)	431 (452)	
SEP (0)			
Low	3,932 (32)	447 (473)	*F =* 0.33*, p =* .718
Medium	5,062 (42)	439 (459)	
High	3,194 (26)	443 (441)	
Location (0)			
Rural/Remote	4,347 (36)	487 (447)^*a*^	*t =* 7.76*, p <* .001
Metropolitan	7,841 (64)	420 (446)^*b*^	
Cultural Background (148)			
English	11,052 (91)	451 (459)^*c*^	*F =* 17.96*, p <* .001
European	208 (2)	508 (482)^*d*^	
Asian	703 (6)	312 (426)^*a*^	
Middle-Eastern	110 (1)	381 (503)^*b*^	
Other	97 (1)	544 (554)^*d*^	
BMI Category (0)			
Underweight	587 (5)	322 (404)^*a*^	*F =* 15.36*, p <* .001
Healthy Weight	8,831 (72)	447 (456)^*b*^	
Overweight	2,219 (18)	464 (484)^*b*^	
Obese	551 (5)	432 (460)^*b*^	
PA Guidelines (0)			
Meeting	1,872 (15)	769 (568)^*a*^	*t =* 34.98*, p <* .001
Not Meeting	10,316 (85)	435 (462)^*b*^	
Screen-Based Entertainment Guidelines (38)			
Meeting	2,395 (20)	476 (449)^*a*^	*t =* 3.85*, p <* .001
Not Meeting	9,755 (80)	435 (462)^*b*^	
Fruit and Vegetable Guidelines (79)			
Meeting	1,762 (15)	613 (528)^*a*^	*t =* 17.09*, p <* .001
Not Meeting	10,341 (85)	413 (440)^*b*^	
Sugar-Sweetened Beverages (116)			
Daily or more often	1,629 (13)	481 (507)^*a*^	*t =* 3.65*, p <* .001
Less than daily	10,443 (87)	436 (450)^*b*^	
High-Fat Food (153)			
Weekly or more often	5,173 (43)	456 (475)^*a*^	*t =* 2.80*, p =* .005
Less than weekly	6,863 (57)	432 (445)^*b*^	

Note. ^1^OSP = Organised Sports Participation and is reported in minutes per week, ^*abc*^ represents significant differences between groups ^*a*^, ^*b*^, ^c^ and ^*d*^.

### Patterns of organized sports participation

Mean levels of participation are outlined by demographic and outcome variables in Table 1. The mean (±SD) weekly participation in organized sports was 443 ± 459 minutes. Participation was significantly higher among males and among those living in rural/remote areas, but did not differ by age or SEP. Differences by cultural background were evident, with those whose primary language was Asian reporting lower participation in organized sports than those whose primary language was English or European. Those whose primary language is Middle-Eastern also had a lower mean level of organized sports participation than English and European speakers. Those categorized as underweight had the lowest levels of organized sports participation with no differences among healthy weight, overweight, or obese students. Those who complied with physical activity, electronic screen time, and fruit and vegetable guidelines reported significantly higher mean levels of participation in organized sports. Students who more frequently consumed sugar-sweetened beverages or high-fat foods also had significantly higher mean levels of organized sports participation.

### Organized sports participation and compliance with physical activity and screen-based entertainment guidelines

Those in quintiles 4 and 5 of organized sports participation (>406 min.week^-1^) were 2.07 and 5.26 times more likely to comply with physical activity guidelines, respectively, than those who reported participating in ≤60 min.week^-1^ (Table 2). Students who reported >210 min.week^-1^ of organized sports participation (quintiles 3–5) were 1.42-1.48 times more likely to comply with national screen-based entertainment guidelines than those who reported ≤60 min.week^-1^.

**Table 2:** **Unadjusted**^**^ **^**and adjusted**^**# **^**odds ratios associated with quintiles of organised sports participation in minutes per week**

**Unadjusted Odds Ratios [95% CI]**														
**Quintile**	**Physical activity guidelines**		**Screen guidelines**		**Fruit and vegetable guidelines**		**Sugar-sweetened beverages consumed daily**		**High-fat food consumed weekly**		**Overweight or Obese**		**Obese**	
	**OR [95% CI]**	***p***	**OR [95% CI]**	***p***	**OR [95% CI]**	***p***	**OR [95% CI]**	***p***	**OR [95% CI]**	***p***	**OR [95% CI]**	***p***	**OR [95% CI]**	***p***
**1**	Reference	-	Reference	-	Reference	-	Reference	-	Reference	-	Reference	-	Reference	-
**2**	1.02 [0.79–1.33]	.003	1.09 [0.86–1.38]	.470	1.05 [0.83–1.33]	.684	0.92 [0.76–1.17]	.425	0.91 [0.81–1.03]	.134	0.89 [0.76–1.05]	.159	0.79 [0.57–1.10]	.161
**3**	1.43 [1.13–1.82]**	.000	1.45 [1.18–1.80]**	.001	1.37 [1.10–1.72]**	.006	0.95 [0.77–1.17]	.632	0.87 [0.75–1.02]	.100	0.90 [0.75–1.08]	.249	0.75 [0.47–1.19]	.226
**4**	2.49 [2.00–3.10]**	.000	1.42 [1.12–1.71]**	.004	1.65 [1.34–2.04]**	.000	0.91 [0.73–1.13]	.398	1.07 [0.93–1.23]	.337	0.88 [0.75–1.02]	.094	0.80 [0.59–1.10]	.168
**5**	6.37 [5.13–7.90]**	.000	1.38 [1.11–1.71]**	.003	2.98 [2.49–3.55]**	.000	1.15 [0.92–1.44]	.193	1.16 [0.93–1.44]	.558	1.09 [0.93–1.27]	.288	0.97 [0.60–1.55]	.888
**Adjusted Odds Ratios [95% CI]**														
**1**	Reference	–	Reference	–	Reference	–	Reference	–	Reference	-	Reference	-	Reference	-
**2**	0.93 [0.73–1.20]	.583	1.08 [0.85–1.38]	.514	1.00 [0.78–1.27]	.984	0.91 [0.75–1.11]	.355	0.92 [0.82–1.05]	.219	0.91 [0.78–1.06]	.234	0.83 [0.59–1.16]	.278
**3**	1.20 [0.94–1.52]	.135	1.48 [1.19–1.84]**	.000	1.32 [1.05–1.67]**	.018	0.88 [0.71–1.09]	.253	0.86 [0.73–1.01]	.065	0.92 [0.77–1.10]	.364	0.79 [0.49–1.27]	.337
**4**	2.07 [1.67–2.58]**	.000	1.45 [1.14–1.85]**	.003	1.60 [1.29–1.98]**	.000	0.83 [0.66–1.03]	.091	1.03 [0.89–1.19]	.674	0.88 [0.76–1.04]	.150	0.83 [0.59–1.15]	.262
**5**	5.26 [4.29–6.44]**	.000	1.42 [1.14–1.76]**	.002	2.90 [2.41–3.49]**	.000	1.02 [0.81–1.28]	.883	0.99 [0.85–1.14]	.843	1.08 [0.93–1.26]	.314	0.97 [0.61–1.53]	.891

*p < .01, **p < .001

^Analyses were adjusted for clustering at the school level, and school type.

^#^Adjusted for sex, age, SEP, home location, cultural background, school level clustering and school type.

### Organized sports participation and eating behaviors

Students who reported >210 min.week^-1^ of organized sports participation were between 1.32 (Quintile 3) and 2.90 (Quintile 5) times more likely to report compliance with national fruit and vegetable consumption guidelines when compared with those who reported ≤60 min.week^-1^ (Table 2). There was no difference in likelihood of consuming sugar-sweetened beverages daily or high-fat foods weekly based on quintiles of organized sports participation.

### Organized sports participation and weight status/adiposity

There was no difference in likelihood to be overweight/obese, or obese, across quintiles of organized sports participation (Table 2). Adjusted multiple linear regressions revealed no association between organized sports participation and waist-to-height ratio (*t* = 1.40, *p* = 0.163), but a small positive relationship with waist circumference (β *=* 0.0006, *t* = 2.75, *p* = 0.006). This equated to a 3.6 mm increase in waist circumference for every additional 10 hours of organized sports participation per week.

## Discussion

This study examined associations between organized sports participation, adiposity and key obesity-related behaviors, using a nationally-representative sample of Australian secondary students. Consistent with previous international research, males spent more time in organized sports than females [29,30]. In contrast, no differences were observed across neighbourhood levels of SEP. This differs from national data showing that nonparticipation in organized sports is higher amongst families of a lower household level SEP [12]. There may be key differences between neighbourhood and household level variables in predicting children’s obesity-related behaviours [31]; therefore, more research is needed in this area to make reliable conclusions regarding potential differences in weekly minutes of participation by levels of SEP. Those in rural and remote communities reported higher levels of participation than those in metropolitan locations. This is also in contrast to national data that reports higher participation rates among capital city dwellers [12]. It is possible that rural and remote areas provide a safer environment in which to participate in sports [32] or be conducive to longer periods of participation than urban areas.

Those of Asian and Middle-Eastern backgrounds reported significantly fewer minutes spent in organized sports per week. This is consistent with national participation rates among younger adolescents whereby lower levels of participation are reported by adolescents of non-English speaking backgrounds [12]. This is a promising avenue of future research given that organized sport has been used to promote health among non-English speaking Australians [33].

There were no differences in sports participation by weight status. This suggests that organized sports are a viable physical activity option for overweight and obese adolescents. There were, however, low levels of organized sports participation among underweight adolescents. This is consistent with previous Australian findings which demonstrate that underweight adolescents expend less energy through daily sports participation than their healthy-weight peers [34]. This may be due to low levels of muscle mass and subsequently decreased opportunities for success in competitive sports. Therefore, underweight adolescents may elect not to participate in organised sports.

Higher rates of sports participation were associated with an increased likelihood of complying with national physical activity guidelines. This is consistent with a large body of previous literature that demonstrates a positive association between participation in sports and levels of physical activity [10], and affirms national and international policies to promote organized sports to combat the burden of physical inactivity [5-7]. Higher levels of sports participation were also associated with an increased likelihood of complying with electronic screen time guidelines. This is an important finding given the dearth of research in this area. Sirard et al. [35] have also reported that participation in team sports had a negative dose–response relationship with television viewing amongst U.S. adolescents. It may be that participation in organized sports takes place at times when opportunities for screen-based behaviors are also high, such as in the after-school hours. This is an interesting avenue of research because physical activity and screen-based behaviors are independent behaviors that do not necessarily correlate in adolescents [36]. While more research is needed to investigate whether increasing sports participation can play a causal role in reducing harmful sedentary behaviours such as television viewing, these findings suggest that policies to combat physical inactivity through the promotion of organized sports in Australia may be justifiably extended to the public health goals of reducing sedentary behaviors.

We found that those who participated in >210 minutes of organized sports per week were more likely to meet national fruit and vegetable consumption guidelines, with a greater likelihood associated with higher quintiles of participation. This is consistent with a recent systematic review [10]; however, in contrast to this review we found no association between sports participation and the likelihood to frequently consume sugar-sweetened beverages or high-fat foods in our adjusted models. Unadjusted means (Table 1) showed that those who regularly consumed high-fat foods and SSB had significantly higher levels of sports participation than those who were irregular consumers. However, these differences were no longer significant when the models were adjusted for covariates. This is a particularly interesting finding. Given the typical clustering of health-related behaviors such as physical activity and healthy dietary behaviors [37] it could be expected that organized sports participation would be associated with both increased fruit and vegetable consumption and decreased consumption of high-fat foods and sugar-sweetened beverages. The lack of association with unhealthy dietary behaviors may be due to several factors. Despite the relatively small contribution of sports food outlets to overall calorie intake, the provision and promotion of unhealthy food choices at sports events is extremely common, and sends contradictory messages regarding acceptable food choices which lead to unhealthy dietary behaviors [10]. Alternatively, the consumption of high-fat foods and sugar-sweetened beverages may be so pervasive in the population that measures of organized sports participation are unable to differentiate between those who do and do not consume these unhealthy options on a regular basis.

We found that sports participation was not associated with weight status, but was associated with waist circumference. However, this positive association was very small, and was potentially due to low levels of organized sports participation among underweight adolescents, or the finding that regular consumers of sugar-sweetened beverages and high fat foods participated in more organized sports than irregular consumers (in unadjusted analyses). Overall, however, it may be concluded that participation in organized sports has little relationship with weight status or adiposity, and this conclusion would be consistent with findings from a systematic review [10]. There are two plausible explanations for this. Firstly, organized sports are not solely for individuals of a healthy weight. National studies conducted in the U.S. concluded that more than one in four organized sports participants are overweight or obese, while more than half of youth who are obese report participating in organized sports [38,39]. Alternatively, because the value of sports to obesity prevention is primarily due to their contribution to total physical activity, low levels of physical activity during organized youth sports [40] may in part explain the lack of association with weight status.

This study provides representative data on duration of sports participation among Australian adolescents that is consistent with national estimates [12]. The dataset also included a valid measure of participation in organized sport in minutes per week, without which the accurate estimation of the health benefits of sports at a population level would not be possible. Further, the sample size is sufficiently large to detect differences in behavioral risk factors according to the level of sports participation. The addition of measures of waist circumference and waist-to-height ratio to a measure of BMI adds strength to the conclusions regarding sports participation and weight status.

Limitations include the reliance on self-report measures of participation in physical activity, screen time and eating habits. In particular, concurrent self-reported participation in organized sports and physical activity are likely to be strongly related. Furthermore, SEP has been measured using participants’ postcode and not at an individual or familial level and may not accurately reflect their ability to afford organized sports or physical activity opportunities. The cross-sectional nature of the study limits assertions of causality. It may be that those adolescents who are more active and have healthier eating habits are more likely to participate in organized sports.

## Conclusions

Participation in organized sports is associated with a greater likelihood to engage in a cluster of beneficial obesity-related health behaviors, including meeting physical activity guidelines, electronic screen time recommendations, and fruit and vegetable consumption guidelines. However, in this study participation in organized sports was not associated with other food habits including the consumption of sugar-sweetened beverages and high-fat foods, nor was it associated with a reduced likelihood to be overweight or obese. These findings suggest that current organized sports programs for Australian adolescents are not associated with lower levels of adiposity, and their role in promoting energy balance is unclear. The association between time spent participating in organized sports and some healthy obesity-related behaviors among Australian adolescents is promising, however, causation needs to be investigated using experimental research. In particular, future research should investigate the role of organized sports in facilitating health for adolescent girls, underweight adolescents, and adolescents of non-English speaking backgrounds for whom this study has shown that organized sports participation is low.

## Abbreviations

APARQ: Adolescent physical activity recall questionnaire; BMI: Body mass index; NaSSDA: National secondary students’ diet and activity survey; SEIFA: Socio-economic indexes for areas; SEP: Socio-economic position; STROBE: Strengthening the reporting of observational studies in epidemiology.

## Competing interests

The authors have no competing interests to disclose.

## Authors’ contributions

SV conceptualized and designed the study, carried out the initial analyses, drafted the initial manuscript, and approved the final manuscript as submitted. DC conceptualized and designed the study, reviewed and revised the manuscript, and approved the final manuscript as submitted. AO conceptualized and designed the study, reviewed and revised the manuscript, and approved the final manuscript as submitted. MS coordinated and supervised use and analyses of all data, reviewed and revised the manuscript, and approved the final manuscript as submitted. BM coordinated and supervised use and analyses of all data, critically reviewed the manuscript, and approved the final manuscript as submitted.

## Authors’ information

SV is a postdoctoral research associate at the Interdisciplinary Educational Research Institute, University of Wollongong. DC is a postdoctoral research fellow at the Interdisciplinary Educational Research Institute, University of Wollongong. AO is a Professor at the Interdisciplinary Educational Research Institute, University of Wollongong. MS and BM are both with the Centre for Behavioural Research in Cancer, Cancer Council Victoria.
